# Reflecting on Progress of the *Malaysian Journal of Medical Sciences*: 2023–2024

**DOI:** 10.21315/mjms2024.31.6.1

**Published:** 2024-12-31

**Authors:** Siti Nor Qamariah Ismail, Jafri Malin Abdullah

**Affiliations:** 1 Production Editor, Malaysian Journal of Medical Sciences, Universiti Sains Malaysia, 11800 USM, Pulau Pinang, Malaysia; 2 Chief Editor, Malaysian Journal of Medical Sciences, Universiti Sains Malaysia Health Campus, 16150 Kubang Kerian, Kelantan, Malaysia

**Keywords:** journal performance report, submission trend, medical sciences journal

## Abstract

This editorial reviews the *Malaysian Journal of Medical Sciences*’ performance over 2023–2024, highlighting key achievements and challenges. It aims to provide a detailed analysis of the journal’s processes and identify areas for improvement.

## Introduction

As we conclude another year, it is imperative to reflect on the progress and challenges encountered by the *Malaysian Journal of Medical Sciences* (MJMS). This editorial provides a comprehensive overview of the journal’s performance for the period 2023–2024. The objective is to offer detailed insights into our processes and to identify areas for potential improvement. Data were collected from 1 January 2023 to 20 December 2024.

### Manuscript Submissions

The submission trend of new manuscripts in 2023 exhibited a continual decline of 2.60% (1). However, in 2024, there was a notable reversal in this trend, with submissions increasing by 32.30%, from 675 to 893 manuscripts.

**Figure 1:**
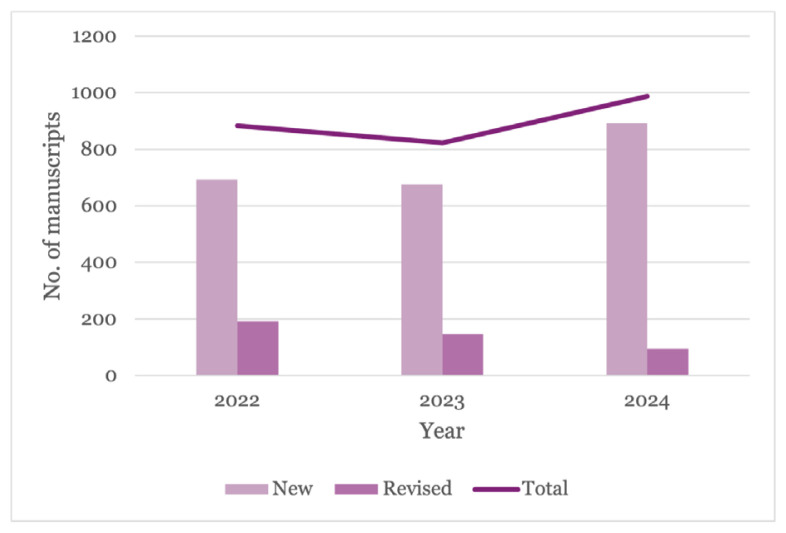
Number of manuscripts submitted to MJMS in 2022–2023 Source: https://mc.manuscriptcentral.com/maljms

Figure 1 presents the number of new, revised, and total manuscript submissions over three years: 2022, 2023, and 2024. Overall, the data shows a decline in both new and revised submissions from 2022 to 2023, followed by a notable increase in new submissions in 2024, leading to the highest total number of submissions in the three-year period.

### Submission Pattern by Manuscript Type

**Figure 2:**
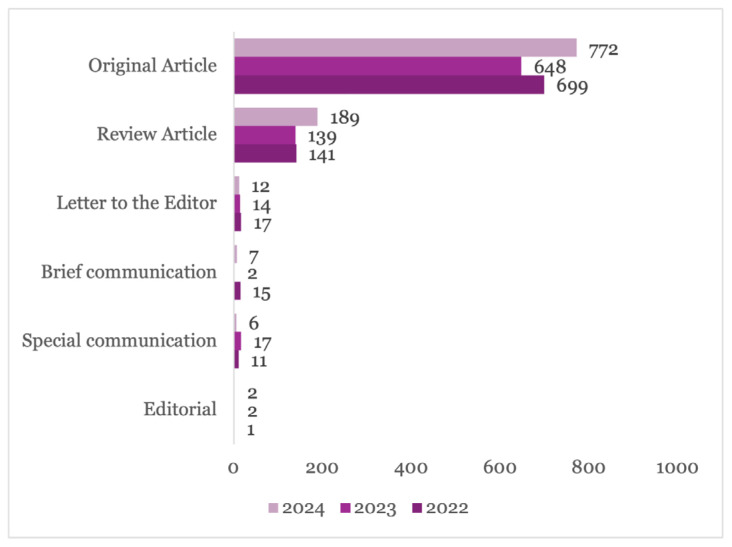
Number of submissions by manuscript type Source: https://mc.manuscriptcentral.com/maljms

Figure 2 provides a comprehensive breakdown of the types of manuscripts submitted from 1 January 2022 to 20 December 2024. The data indicates that submissions of Original Articles, which constitute the major type of manuscript submitted, decreased from 699 in 2022 to 648 in 2023, representing a decline of 7.30%. However, in 2024, there was a significant increase in submissions, rising by 19.14% to reach 772.

Similarly, Review Articles, the second largest type of manuscripts submitted, experienced a slight decrease in submissions, from 141 in 2022 to 139 in 2023, representing a decline of 1.42%. However, in 2024, submissions in this category saw a notable increase, rising by 35.97% to 189.

Submissions of Brief Communication manuscripts dropped significantly from 15 in 2022 to 2 in 2023. In 2024, submissions in this category increased to 7. Special Communication manuscript submissions increased from 11 in 2022 to 17 in 2023. However, in 2024, submissions in this category decreased to 6. It is important to note that some editorial articles are solicited and therefore, not reported through the ScholarOne system.

Figure 2 reveals fluctuations in the number of submissions across different manuscript types, with a significant overall increase in 2024, particularly for Original Articles and Review Articles.

### Submission of Manuscripts by Month and Year

**Figure 3:**
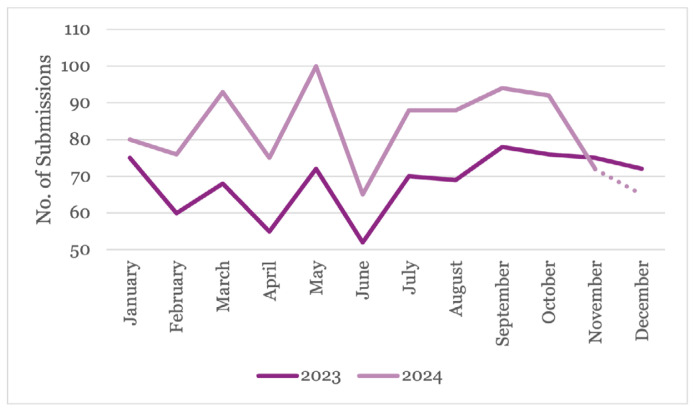
Number of submissions by months in 2023 and 2024 Source: https://mc.manuscriptcentral.com/maljms

Figure 3 indicates a positive trend in manuscript submissions in 2024, with substantial increases in most months compared to 2023. The most significant increases in submissions were observed in May (38.89%), March (36.76%), and April (36.36%). Interestingly, manuscript submissions decreased during the publication months (February, April, June, August, October, and December). However, they fluctuated back to an increase during non-publication months, except in the final quarter of both years.

### Submission of Manuscripts by Country

**Figure 4:**
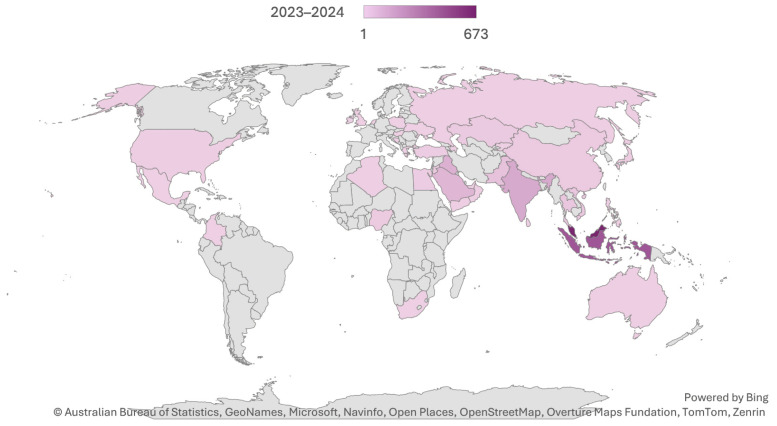
Distributions of manuscript submissions by country for 2023–2024

Table 1 presents the number of manuscript submissions by region and country for the years 2023 and 2024. It highlights the contributions from various countries, showing trends and changes over the two-year period. This data helps identify regions with significant increases or decreases in submissions, providing insights into the geographical distribution of manuscript contributions, as visualised in Figure 4.

### Manuscripts Final Decision

Table 2 highlights the changes in the acceptance and rejection ratios over the two years, providing insights into the editorial decisions. The rejection rate increased from 87.2% in 2023 to 92.2% in 2024, suggesting a more stringent review process and a potential decline in the quality of submissions received.

### Duration from Submission to Final Decision

The average time from submission to final decision has improved over the past two years. In 2023, it was 46.50 days; in 2024, it decreased to 40.35 days. This reduction reflects efforts by editorial boards to streamline review processes and increase efficiency.

### Duration form Acceptance to Publication

The average time from acceptance to publication has shown a notable improvement over the past two years. In 2023, the process took an average of 10 months. However, by 2024, this duration had significantly decreased to 7 months.

### Journal Metrics

The journal impact factor for MJMS in 2023 is 1.1, placing it in the fourth quartile (Q4) within the Medicine, Research and Experimental category, where it ranks 152nd out of 189 journals.

In terms of Scopus indexation, the journal’s CiteScore has shown improvement, increasing from 2.6 in 2022 to 2.7 in 2023. This improvement has elevated its rank from 322nd out of 830 journals (61st percentile) in the Medicine (General Medicine) category in 2022 to 198th out of 636 journals (68th percentile) in 2023. These metrics indicate a positive trend in the journal’s citation performance and its growing influence within the academic community.

### New Members of the Editorial Board

MJMS is pleased to welcome Associate Professor Dr. Rafidah Hanim binti Shueb @ Shomiad from Universiti Sains Malaysia to the Editorial Board. She has been appointed as a laboratory-based editor, effective 1 December 2024.

### Achievements

In 2024, MJMS received the Crème Journal Recognition Award at the Ministry of Higher Education (KPT) – Elsevier Excellence Awards Ceremony and the Crème Journal Recognition in conjunction with the Putrajaya Festival of Ideas on 28 November 2024.

Additionally, at the 15th MAPIM – KPT Awards on 18 December 2024, MJMS won the Best Journal Article Award in Science, Technology, and Medicine (International Index), securing first place. The awarded article, titled “The Antioxidant Activity and Induction of Apoptotic Cell Death by *Musa paradisiaca* and *Trigona* sp. Honey Jelly in ORL115 and ORL188 Cells,” was authored by Mohd Nur Nasyriq Anuar, Muhammad Ibrahim, Nor Hafizah Zakaria, Solachuddin Jauhari Arief Ichwan, Muhammad Lokman Md Isa, Nur Aizura Mat Alewi, Abdullah Hagar, and Fadzilah Adibah Abdul Majid (2).

## Figures and Tables

**Table 1:** Number of submissions by country from 1 January 2023 to 20 December 2024

Region	Country	2023	2024
Africa	Nigeria	11	9
	Egypt	7	7
	Algeria	2	2
	South Africa	0	1

Asia	Malaysia	344	329
	Indonesia	203	259
	Iraq	30	83
	India	63	79
	Saudi Arabia	30	64
	Pakistan	22	30
	Thailand	7	25
	Viet Nam	12	16
	China	6	13
	Kazakhstan	5	11
	The Islamic Republic of Iran	30	8
	Jordan	10	7
	Turkey	7	6
	United Arab Emirates	1	4
	Philippines	0	4
	Oman	1	3
	Brunei Darussalam	9	2
	Japan	7	2
	Yemen	1	2
	Azerbaijan	1	1
	Kyrgyzstan	0	1
	Hong Kong	1	0
	Sri Lanka	1	0
	Taiwan, Province of China	1	0

Europe	Russian Federation	1	2
	The United Kingdom of Great Britain and Northern Ireland	1	2
	Greece	0	2
	Hungary	0	2
	Bosnia and Herzegovina	0	1
	Ireland	0	1
	Ukraine	0	1
	Netherlands	1	0
	Poland	1	0

Oceania	Australia	1	4

North America	United States	4	3
	Mexico	0	1
	Trinidad and Tobago	1	0

South America	Colombia	0	1

**Table 2:** Accept and reject ratio from 1 January 2024 to 20 December 2024

Manuscript decision	2023		2024	

	Number of manuscripts	Ratio (%)	Number of manuscripts	Ratio (%)
Accept	85	12.8	61	7.8
Reject	578	87.2	726	92.2

Total	663	100.0	787	100.0
